# Supplementary material for: Elementary Classroom Views of Nature Are Associated with Lower Child Externalizing Behavior Problems
Source: Int J Environ Res Public Health. 2023 Apr 26;20(9):5653. doi: 10.3390/ijerph20095653 (PMC10177887; doi:10.3390/ijerph20095653)
Supplement: Supplementary file 1 [file ijerph-20-05653-s001.zip › ijerph-2272144-supplementary.pdf]

Supplementary Materials

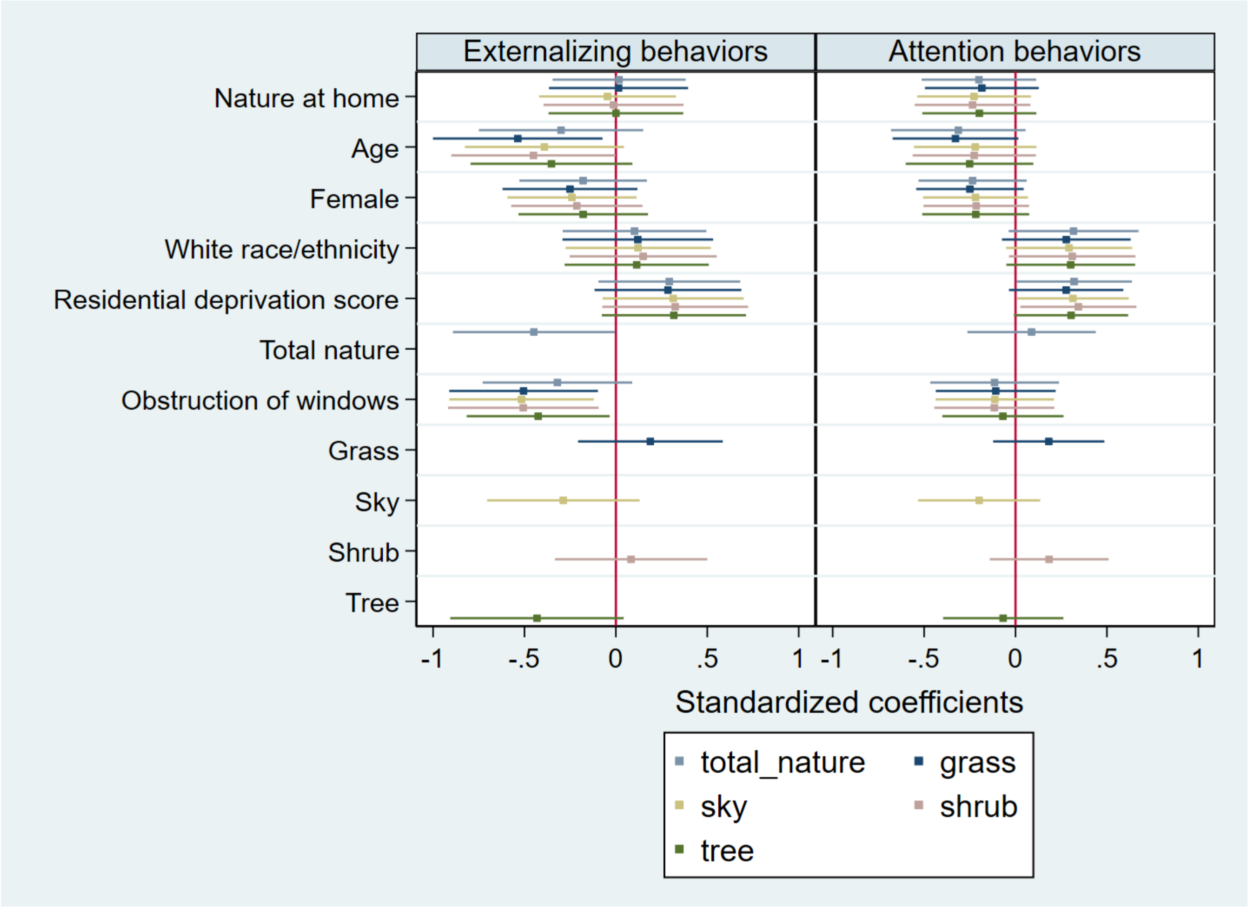

Figure S1. Sensitivity analysis- regression results, adjusting for window obstructions.

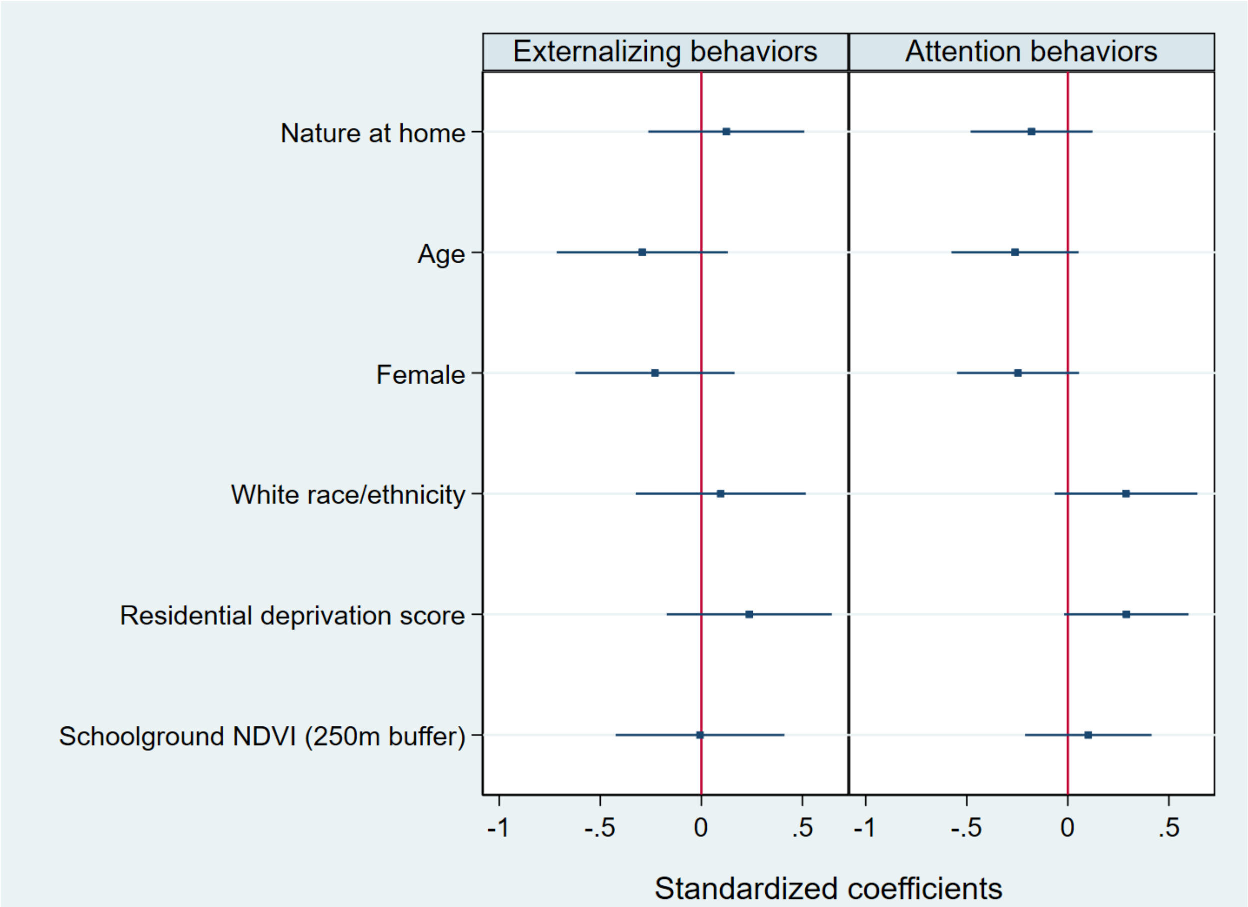

Figure S2. Sensitivity analysis- regression results, schoolground NDVI as the independent variable of interest

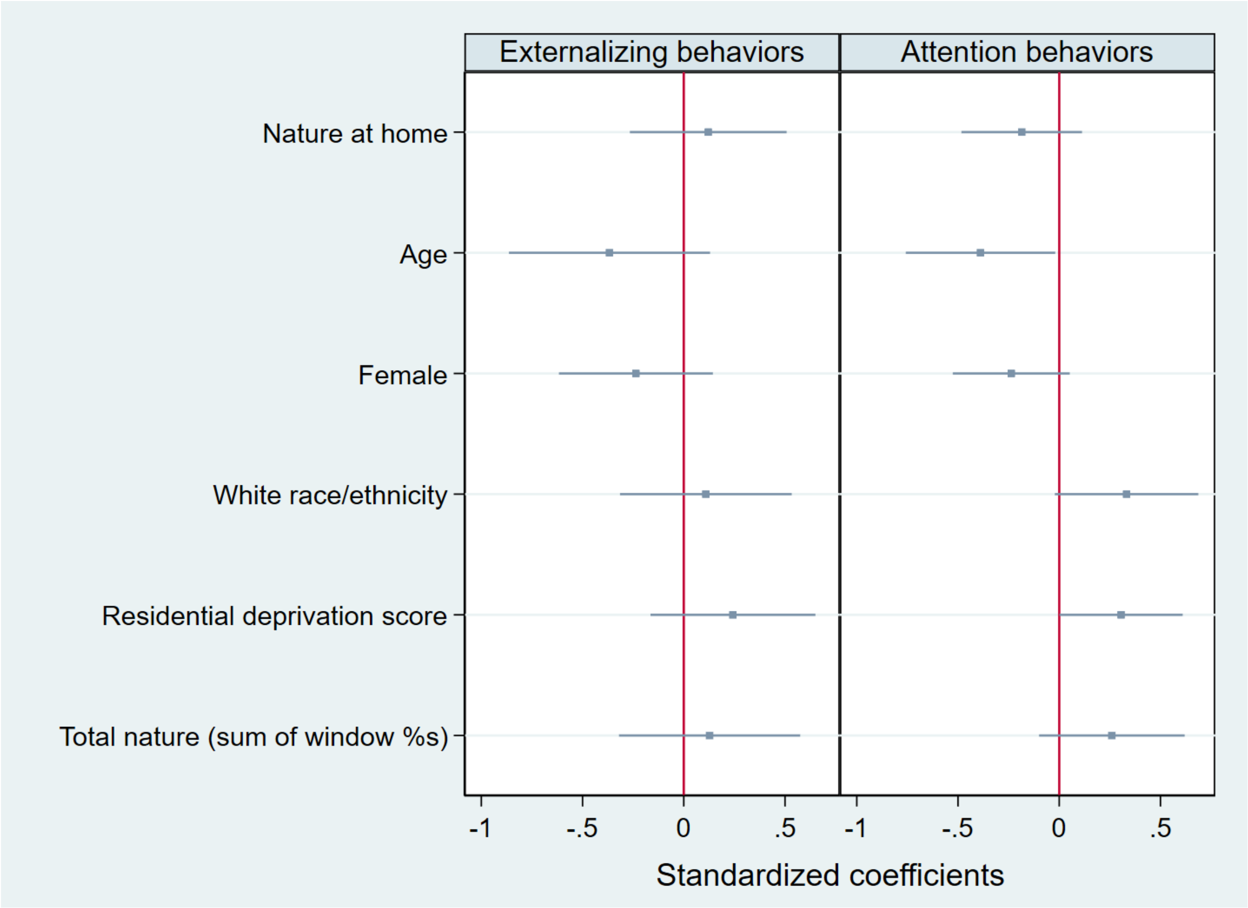

Figure S3. Sensitivity analysis- regression results, sum of each window's % nature as the independent variable of interest

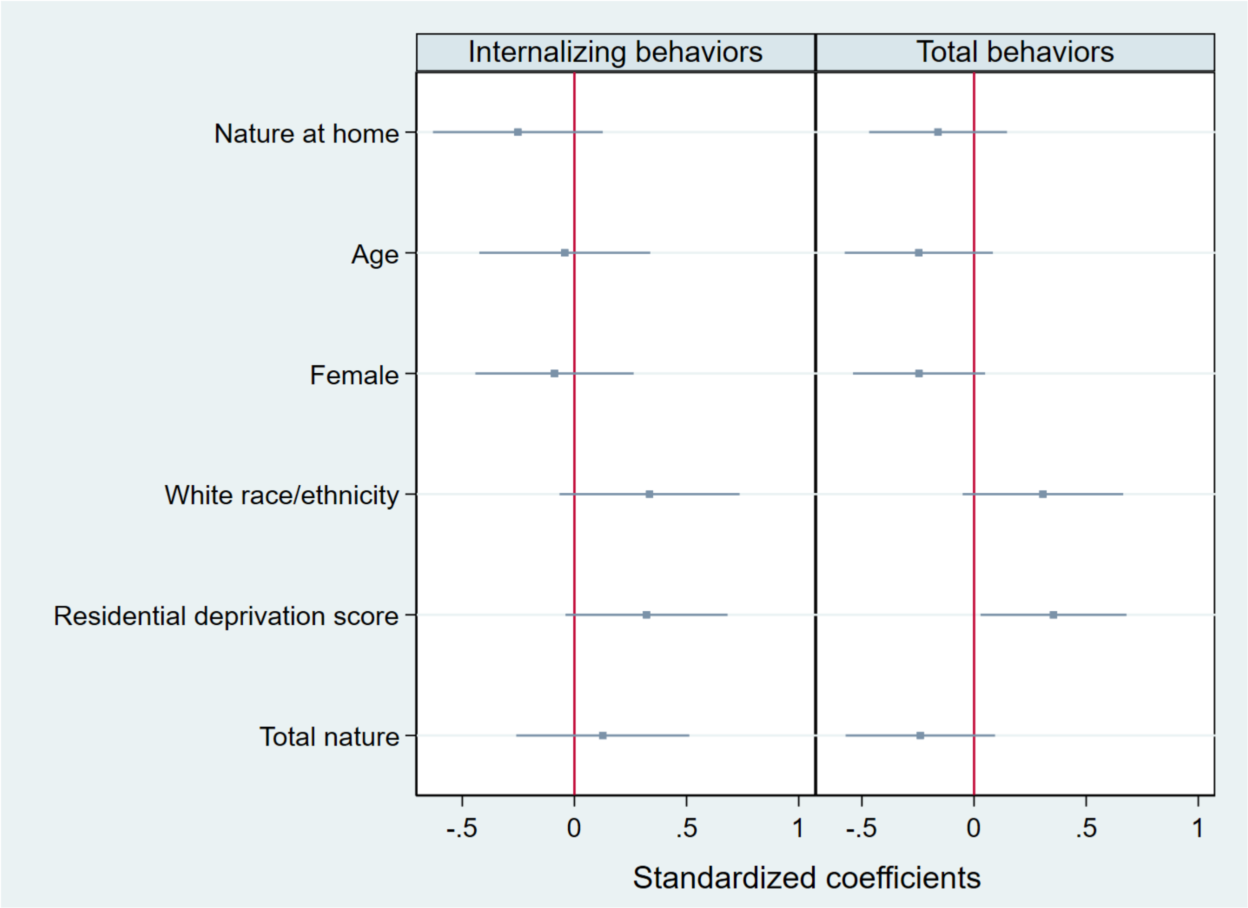

Figure S4. Sensitivity analysis- regression results for predicting internalizing behaviors (5 items) and total behaviors (18 items) (exploratory only)

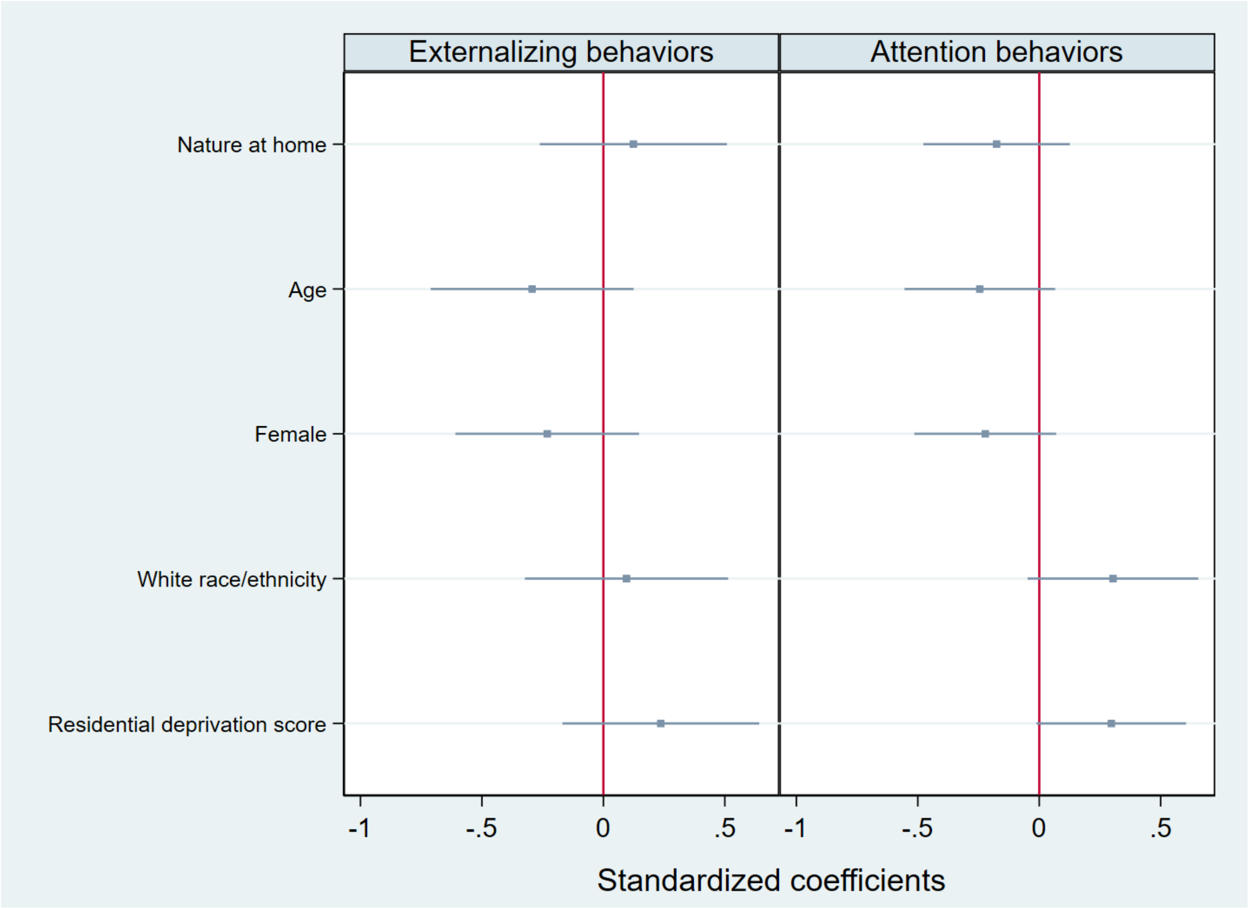

Figure S5. Sensitivity analysis- regression results for nature at home as the independent variable of interest
